# LC-MSsim – a simulation software for liquid chromatography mass spectrometry data

**DOI:** 10.1186/1471-2105-9-423

**Published:** 2008-10-08

**Authors:** Ole Schulz-Trieglaff, Nico Pfeifer, Clemens Gröpl, Oliver Kohlbacher, Knut Reinert

**Affiliations:** 1 International Max Planck Research School for Computational Biology and Scientific Computing, Berlin, Germany; 2 Department Computer Science and Mathematics, Free University of Berlin, Berlin, Germany; 3 Wilhelm Schickard Institute for Computer Science, Tübingen University, Tübingen, Germany

## Abstract

**Background:**

Mass Spectrometry coupled to Liquid Chromatography (LC-MS) is commonly used to analyze the protein content of biological samples in large scale studies. The data resulting from an LC-MS experiment is huge, highly complex and noisy. Accordingly, it has sparked new developments in Bioinformatics, especially in the fields of algorithm development, statistics and software engineering. In a quantitative label-free mass spectrometry experiment, crucial steps are the detection of peptide features in the mass spectra and the alignment of samples by correcting for shifts in retention time. At the moment, it is difficult to compare the plethora of algorithms for these tasks. So far, curated benchmark data exists only for peptide identification algorithms but no data that represents a ground truth for the evaluation of feature detection, alignment and filtering algorithms.

**Results:**

We present *LC-MSsim*, a simulation software for LC-ESI-MS experiments. It simulates ESI spectra on the MS level. It reads a list of proteins from a FASTA file and digests the protein mixture using a user-defined enzyme. The software creates an LC-MS data set using a predictor for the retention time of the peptides and a model for peak shapes and elution profiles of the mass spectral peaks. Our software also offers the possibility to add contaminants, to change the background noise level and includes a model for the detectability of peptides in mass spectra. After the simulation, *LC-MSsim *writes the simulated data to mzData, a public XML format. The software also stores the positions (monoisotopic m/z and retention time) and ion counts of the simulated ions in separate files.

**Conclusion:**

*LC-MSsim *generates simulated LC-MS data sets and incorporates models for peak shapes and contaminations. Algorithm developers can match the results of feature detection and alignment algorithms against the simulated ion lists and meaningful error rates can be computed. We anticipate that *LC-MSsim *will be useful to the wider community to perform benchmark studies and comparisons between computational tools.

## Background

In mass spectrometry (MS) based proteomics, proteins in a sample are digested and the resulting peptides are separated by high-performance liquid chromatography (LC) before injecting them into the mass spectrometer [1]. In this work, we focus on data from LC-MS experiments, as opposed to LC-MS/MS experiments where a fragmentation of selected sample compounds is performed to obtain ion ladders which can be used for the identification of the compound [2]. Pure LC-MS experiments do not directly give information about the sequences of the peptides in a sample but we can still use the information on the LC-MS level to perform a quantification of the sample proteins [3]. In this application, algorithms detect peptide ion signals (features) in LC-MS spectra and estimate their abundances by integrating the signal area. Different charge variants of the same peptide are summarized (deconvoluted) and the peptides are mapped back to their parent protein to obtain abundance estimates at the protein level.

**Figure 1:**
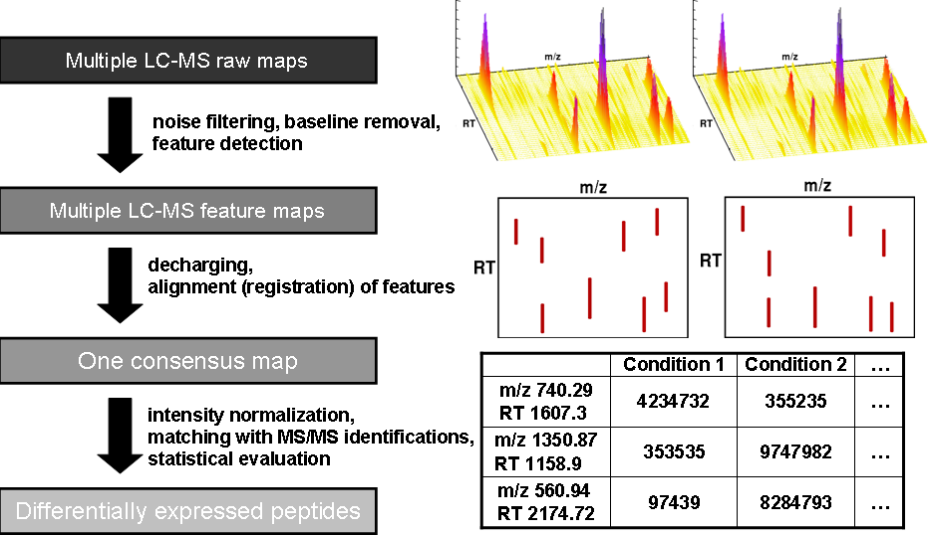
**LC-MS data analysis**. A generic workflow for LC-MS data analysis: This figure illustrates key steps in a typical workflow, which comprise feature detection, spectra alignment and statistical evaluation.

Modern mass spectrometers can easily generate thousands of mass spectra in a short time. This wealth of information has sparked off the development of new, fully automated methods to analyze and process it. Fig. 1 gives an example of a generic data analysis workflow in a study using LC-MS. Stages of mass spectrometry-based proteomics in which algorithms can be applied are, among many others, low-level preprocessing such as the abovementioned feature detection and quantification [4-6], alignment and registration of LC-MS data sets [7-9] as well as the statistical evaluation [10] of the experiments. There also exist some software tools that offer all (or most) of these steps in one program [11-14]. For a recent review, we refer the interested reader to Müller *et al*. [15].

This plethora of tools is often confusing for the user who needs to decide which algorithms to apply for his data. But also developers of new algorithms need standardized benchmark data to compare their approach to existing ones. This is a difficult task, since only few quality metrics [16] and only limited benchmarks exist so far. Carefully compiled databases of annotated test data are standard in other fields such as DNA sequence [17-19] or RNA structure analysis [20]. But they are not yet available for mass spectrometry based proteomics. Only few researchers make their LC-MS data publicly available and all proteomic databases so far focus on data for the identification of peptides from MS/MS spectra [21-25] and not on broader applications such as quantitative experiments.

An ideal LC-MS data set for the evaluation of feature detection, alignment and quantification algorithms would contain annotations with the positions of all peptide ion signals, their charge states, monoisotopic masses and abundances. Only this information would allow meaningful comparisons between different methods and fair benchmark studies. Of course, this information can be partially obtained by peptide identifications using MS/MS fragmentation. Unfortunately, only a few of the peptide ions present in a sample are selected for fragmentation. Furthermore, even of those fragmented, many cannot be identified due to noise, mutations or posttranslational modifications. For these reasons, annotations by MS/MS will always be incomplete. Manual annotations by a human expert have been performed for single data sets [6] but are clearly infeasible if our aim is to generate larger benchmarks. We believe that the simulation of LC-MS spectra is a valid approach, to be supplanted by the accumulation of annotated real-word spectral databases.

In the following sections, we introduce our software *LC-MSsim *and describe its implementation details. We would like to emphasize that our aim was not to create a detailed physical model of mass spectra generation as, for instance, attempted in [26]. But we want to simulate data that is reasonably close to reality and provides a fair testing ground for data analysis methods. The idea of simulating ESI mass spectra to assess the performance of MS feature detection algorithms was pioneered by Wong *et al*. [27] who presented a straightforward model for the simulation of ESI mass spectra. They simulate spectra as mass lists derived from theoretical digests of protein sequences with normalized intensities without prediction of ion intensities, retention times or simulation of isotopic pattern. They also restrict their comparison to their own algorithm which implements a very specific task, the detection of protein-ligands and other macromolecular complexes in mass spectra. Of course, the applications of LC-MSsim are not restricted to feature detection benchmarks. The next obvious step would be to compare alignment algorithms, but even the comparison of a full quantification workflow is an interesting scenario.

To our knowledge, *LC-MSsim *is the first software that models the whole LC-MS data acquisition process and delivers an output (the simulated LC-MS map and the list of peptides and contaminants with m/z and retention time) that can directly be used for the assessment of proteomics algorithms. There are, of course, some programs that simulate individual parts of the LC-MS data acquisition process, such as the estimation of isotopic peak patterns [28,29], the prediction of peptide retention times [30-34] or detectability [35,36]. However, these tools are written in different programming languages and they have different output formats that cannot be easily combined. Therefore, to simulate a full LC-MS run, it is clearly desirable to have all of these tools combined in a single application.

## Methods

*LC-MSsim *is written in C++ as an add-on for OpenMS [37], our software library for computational mass spectrometry. *LC-MSsim *uses OpenMS data structures for file reading, writing and the calculation of isotopic patterns. It is also compatible with The OpenMS Proteomics Pipeline (TOPP) [13] and can readily be integrated into its workflows. This makes it very easy to generate large numbers of simulated data sets and to pipe them directly into a TOPP data analysis pipeline. *LC-MSsim *is compatible with the current OpenMS release version 1.1.

Furthermore, *LC-MSsim *supports the TOPP INI (configuration) file format. This format is XML-based and can be edited using common XML editors or the INIFileEditor supplied with TOPP. *LC-MSsim*, OpenMS and TOPP are all published under the Lesser GNU Public License. The source code can be downloaded from .

An artificial LC-MS data set is generated by the following steps: digestion of proteins, prediction of peptide detectability and retention time, relative abundances of charge states, modeling of isotopic and elution profiles and addition of shot noise to spectra. Key parameters that influence the outcome of the simulation are the minimum accepted peptide detectability which influences the number of theoretical peptides appearing in the LC-MS spectra, mass accuracy and resolution, as well as the Full-Width-At-Half-Maximum (FWHM) of the peptide peaks and the percentage of non-peptide contaminants added by the simulation software. In the following sections, we give an overview of all simulation steps and explain their parameters in more detail.

### Protein Digestion

The user can supply a list of protein sequences in a FASTA file and define their relative abundances in the sequence header. If no abundance is given, we assume that each protein, and thus its peptides, will appear in equal abundances in the mass spectra (apart from effects such as ion suppression etc.). *LC-MSsim *supports only tryptic digests in this version, but new proteases can be added easily by extending the corresponding OpenMS classes by new regular expressions. We can also simulate missed cleavages and self-digestion of the protease.

### Detectability and Retention Time Prediction

After the enzymatic digest of all protein sequences, we need to determine the retention time of each peptide. Pfeifer *et al*. [34] recently introduced the *paired oligo-border kernel *(POBK) for machine learning problems in computational proteomics. Support vector regression [38] using this kernel function yields very accurate retention time predictions while requiring only a small number of training samples. We use the *POBK *for retention time prediction in our simulator. We trained the SVM on the test set of Petritis *et al*. [31] and determined the best parameters using nested cross-validation. The data set consists of 1304 peptide identifications of capillary reversed-phase liquid chromatography runs.

**Figure 2:**
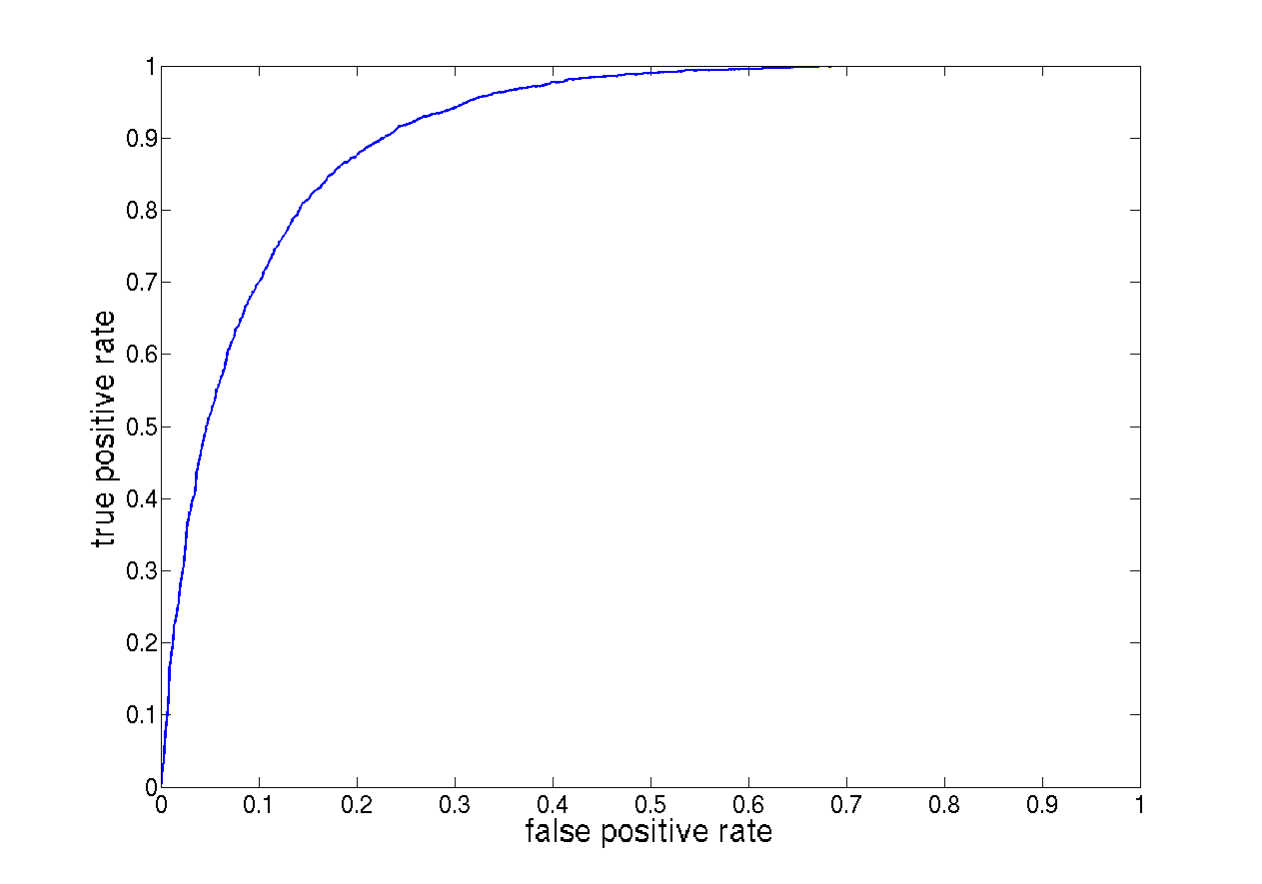
**ROC plot for peptide detectability prediction**. ROC plot for peptide detectability prediction: This plot shows the ROC-curve for peptide detectability prediction on the excluded test partitions of the nested cross-validation runs on balanced samples of the MUDPIT-ESI data set of Mallick *et al*. [35].

It was previously shown that not all peptides of a digested protein actually appear in the LC-MS sample [35,36,39]. There are numerous reasons for that. Some peptides will only poorly ionize in the electrospray and others are simply not soluble, just to name a few. To account for this fact, we determine the likelihood of detectability of each peptide using a support vector machine [40] and the *POBK *as a kernel function. We performed a nested cross-validation on balanced samples of the MUDPIT-ESI data set of Mallick *et al*. [35]. This means that we selected all *n *positive examples and chose *n *negative examples randomly out of all negative examples. The whole process was repeated ten times to minimize random effects. A ROC curve of all predictions on the excluded test partitions can be seen in Fig. 2. Mallick *et al*. [35] evaluated their method in terms of 1 – positive predictive value (PPV) against coverage. For the MUDPIT-ESI data set they got a coverage of about 0.65 at 1 - PPV = 0.1 and a coverage of about 0.75 at 1 - PPV = 0.15. In our evaluation (data not shown) we get a coverage of about 0.65 at 1 - PPV = 0.11 and a coverage of about 0.8 at 1 - PPV = 0.15. This means that our method performs comparable to the methods of Mallick *et al*. [35] although requiring just a fraction of the negative data set for training (about 1000 instead of 25, 000), which drastically decreases the time needed for training the classifier. We used the probability estimates [41] of the libsvm [42] to compute the likelihood of a peptide to appear in the LC-MS spectra.

### Charge Distribution Model

After protein digestion, peptide detectability and retention time prediction, we need to determine the relative abundances of the ions created for each peptide. *LC-MSsim *models an electrospray ionization (ESI) mass spectrometer. ESI ionizes peptides and other sample compounds by applying a strong electric field to the sample. This field induces a charge accumulation at the liquid surface which will form highly charged droplets. As a result, we expect to see one to four ions per peptide, but charge states two or three are the most common. There are several chemical models describing the charge distribution for molecules after ESI and numerous factors influence this distribution such as the pH, sample composition and conformation of the peptide [43,44]. However, our experiments have shown that a simple model gives a good approximation of real data.

For this reason, we decided to stick with a straightforward model of an ESI mass spectrometer in positive ion mode. We follow an approach by Schnier *et al *[45] and assume that each basic amino acid in a peptide can receive at most one charge unit (proton). Consequently, most tryptic peptides have a maximum charge state of 2 – 3 which matches observations of real data. We determine the relative abundances of each charge state by sampling from a binomial distribution. As a result, low charge states are much more likely to occur than higher ones.

### Ion Signal Model

The position of a peptide ion signal in the LC-MS map is determined by three parameters: monoisotopic mass, charge and retention time. We calculate the mass from the amino acid sequence, charge is given by our binomial charge distribution model and the retention time predicted by the SVM.

Usually, a peptide ion gives rise to several peaks in a mass spectrum due to the fact that some of its atoms will occur in heavier isotopic states. Given the sequence of the peptide, we calculate its monoisotopic mass from its empirical formula. The relative heights of the isotopic peaks are calculated using a simple but fast algorithm [46]. This algorithm gives us the relative intensities of the isotopic peaks. We model the peak shape using a Gaussian distribution. The user can choose the peak width in terms of the Full-Width-At-Half-Maximum (FWHM). The FWHM of a peak in a mass spectrum is given by the difference of the m/z values at which the ion count equals half of the maximum ion count of this peak. Note that we assume the peak shape to be Gaussian and the FWHM of a Gaussian function is given by $2\sqrt{2\ln2\sigma }$, where *σ *is the standard deviation of the Gaussian.

**Figure 3:**
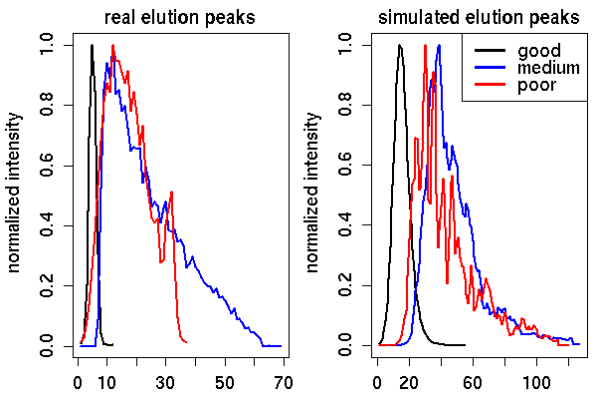
**Comparison of real and simulated elution peaks**. Comparison of real and simulated elution peaks: (Left) Real elution peaks from a Reversed-phase HPLC experiment. (Right) Simulated elution profiles. They represent the three pre-defined column configurations *LC-MSsim *can simulate.

Whereas the shape of peaks in the m/z dimension is relatively stable during one experiment, the peak shape in retention time might vary considerably, but has often a Gaussian-like shape. To account for this fact, we model the elution profile of a peptide signal using different chromatographic functions: a simple Gaussian distribution and an exponentially modified Gaussian distribution (EMG) [47]. Whereas the Gaussian function represents a perfect chromatographic condition, the EMG can model different distortions of the elution peak. Its exponential component introduces tailing and fronting effects. Several studies have shown that it provides a good fit for chromatographic peaks in reverse-phase chromatography [48,49]. It is defined as

$$\text{emg}(x)=\frac{ac\sqrt{2\pi }}{2d}\exp\left(\frac{b-x}{d}\frac{c^{2}}{2d^{2}}\right)\left(\frac{d}{\left|d\right|}-\text{erf}\left(\frac{b-x}{\sqrt{2c}}+\frac{c}{\sqrt{2d}}\right)\right)$$

where *b *is the standard deviation of the Gaussian part, *d *is the expected shift of the exponential modifier, *a *the amplitude and *c *the center. For *d *< 0, we obtain a fronted peak and for *d *> 0, the peak is tailed. Furthermore, we add uniformly distributed noise to single sampling points of the EMG but smooth the noisy elution profile afterwards to obtain ragged chromatographic peaks. This allows us to model more realistic chromatographic peaks as an elution profile in a real LC-MS run is never entirely smooth. On the other hand, this introduces several additional parameters into the simulation. To make the software more user-friendly, we supply a set of pre-defined parameter sets for the EMG, entitled *poor*,*medium *and *good *chromatographic conditions. A choice of good conditions leads to almost perfect Gaussian-shaped peaks, like they will almost never appear in a real experiment. Accordingly, medium and poor conditions lead to far more noisy elution peaks, including tailing and fronting effects. The corresponding peptide signals will be more difficult to trace across all their scans since most algorithms have problems to trace features with very rough elution peaks. Fig. 3 shows three elution peaks from a reversed-phase column and as well as three simulated elution profiles, one for each parameter set. As we can see, the simulated peaks are close to real elution peaks and cover a sufficiently broad range of chromatographic conditions.

**Figure 4:**
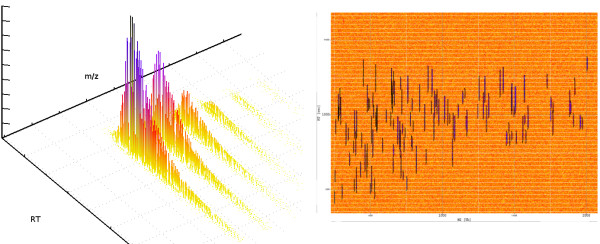
**A simulated LC-MS run**. Bird's eye view of a simulated LC-MS map: A simulated BSA peptide ion signal (left) and a bird's eye view of an LC-MS map of the whole digest (right). The blue signals are the tryptic peptides, red and yellow is shot noise and contaminants.

Putting all this together, we can model LC-MS experiments with different mass resolutions and chromatographic conditions. To exemplify this, the right part of Fig. 4 gives a bird's eye view of an LC-MS map created by *LC-MSsim*. This map represents a tryptic digest of BSA (Bovine Serum Albumin) with some contaminations (metabolites etc., see below). The left part of Fig. 4 shows a simulated BSA peptide ion from this map. This peptide occurs in the charge variant +1 and +2.

### Noise and Contaminations

No real LC-MS data set consists only of true signals i.e. signals caused by sample compounds. There is always some (and often a high amount of) noise in each spectrum. *LC-MSsim *has several parameters that allow the user to introduce noise of various forms into a data set. Users can simulate almost perfect LC-MS runs and runs with high amount of noise posing severe challenges to data analysis algorithms.

First, the user can define error bounds on the theoretically predicted retention times. By doing so, we simulate retention time shifts between different experiments and, for instance, can evaluate the performance of LC-MS alignment algorithms that are used to correct for these shifts as illustrated in Fig. 1. *LC-MSsim *assumes these errors to be Gaussian-distributed and the user can define medium and standard deviation in each case.

Mass analyzers with different mass accuracies and resolutions are simulated by changing the FWHM of the peptide peaks as described above and by altering the sampling step size of the peptide models. Furthermore, *LC-MSsim *simulates inaccuracies in peak intensity measurements by adding Gaussian-distributed noise to peptide peaks. Finally, ESI mass spectra frequently contain high-frequency noise signals of low to medium intensity, often referred to as shot noise. This term stems from electronics and physics [50] and describes statistical fluctuations occurring if the number of particles measured by a detector is very small. Its strength increases with the average intensity of the detected signal but is usually only detectable if the measured signal is very weak. The common assumption is that shot noise is Poisson-distributed [51].

**Figure 5:**
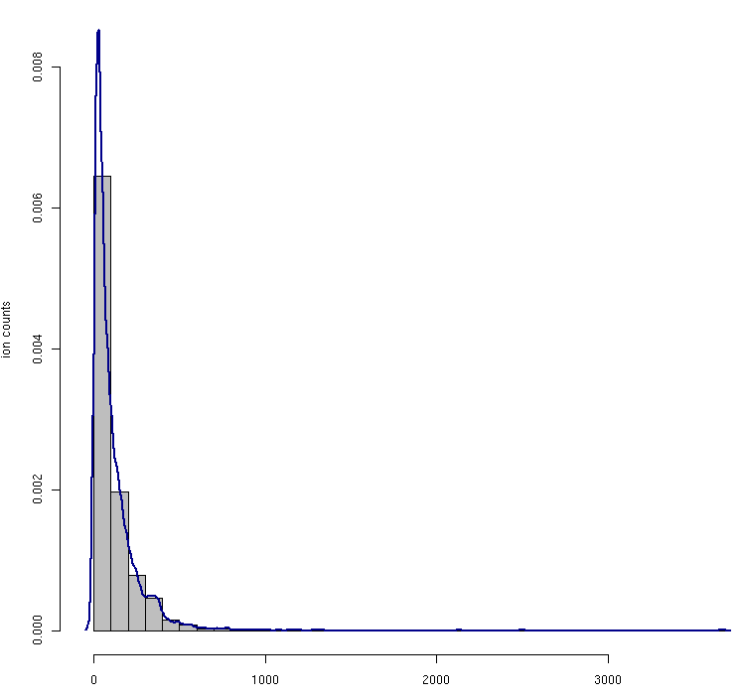
**Intensity distribution of a real mass spectrum**. The intensity distribution (histogram and density plot) of raw data point intensities in a **real **mass spectrum. The plot shows that the true intensities match closely our Poisson-distributed intensity model.

To our knowledge, the notion of shot noise in mass spectrometry is much less well defined than in physics but usually loosely refers to high-frequency noise of low intensity in a mass spectrum. Noise models for mass spectra have been the topic of several publications, but no consensus on the most suitable model exists so far [52-54]. However, recent publications suggests that noise in both Q-TOF and Ion Trap spectra can be modeled using a Poisson distribution [53] and therefore we decided to do the same. We split each spectrum in our simulated LC-MS map into segments of uniform size. We determine the number of shot noise signals by sampling from a Poisson distribution, though m/z and intensity of these particles are given by a Gaussian and Exponential distribution, respectively. Fig. 5 shows the peak intensity distribution of a *real *MS scan. The distribution is approximately exponential with some signals (true peptide peaks) having a high intensity. This shows that our model with exponentially distributed noise intensities well approximates real signals.

Another typical phenomenon in mass spectra is a so-called baseline signal which usually decays with increasing m/z. This is usually a problem for MALDI instruments but less in ESI mass spectrometry. *LC-MSsim *can simulate baseline signals by adding an exponentially-decaying baseline to each mass spectrum, but this feature is turned off by default.

Shot noise and a baseline are both factors that hamper a computational analysis. But of equal concern for feature detection algorithms are non-peptidic contaminations in an LC-MS experiment or peptide signals arising from modified peptides. Hoopmann *et al*. [5] demonstrated that the detection of modified peptides is difficult and requires additional computational effort since the isotopic pattern of these peptides does not follow the typical averagine pattern assumed by most algorithms. In short, an averagine is an average amino acid with a composition estimated from a large number of protein sequences. Using the averagine, we can estimate the average isotopic pattern for a peptide of a given mass. Furthermore, contaminations such as salt molecules or metabolites are of lesser interest in proteomics studies and should not be reported by peptide feature detection algorithms. For these reasons, we decided to simulate these interferences as well. *LC-MSsim *comes with a list of sample contaminants that can easily be extended by editing the corresponding text file. The current list of available contaminants comprises a snapshot of metabolites downloaded from the Human Metabolome Database [55]. The user can set the percentage of added contaminants with respect to the number of peptides.

**Figure 6:**
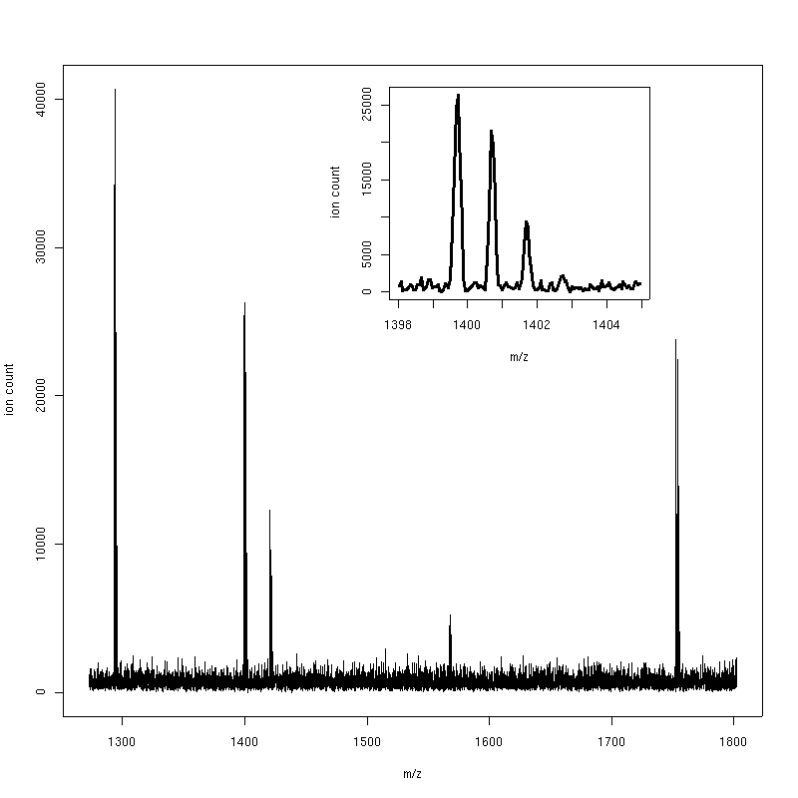
**A simulated mass spectrum**. A simulated mass spectrum of a BSA digest with shot noise added. The inset shows an isotopic pattern from the same scan.

*LC-MSsim *also includes a list of typical modifications such as oxidations or demethylations together with a list of affected amino acid residues. For each peptide containing a matching amino acid, *LC-MSsim *determines at random whether the amino acid is modified or not. The user can set the corresponding probabilities and desired relative frequencies of modified peptides. Fig. 6 shows an MS scan from a simulated BSA digest with added metabolic contaminants and shot noise. It is only a single scan from an LC-MS experiment, therefore not all BSA peptides are visible.

## Results

In this section, we present exemplary applications of *LC-MSsim*. The advantage of our simulator is that we can generate LC-MS maps for which the exact mass, charge, retention time and bounding box of all compounds are known. The bounding box is the smallest axis-parallel rectangle that fully encloses the raw data points constituting the peptide feature. We can also deliberately introduce noise or change instrument parameters such as resolution or chromatographic behavior. This allows scientists to perform fine grained comparisons of LC-MS data analysis algorithms.

We decided to focus on peptide feature detection algorithms and compare the algorithms msInspect [11], Superhirn [15], SpecArray [56], MZmine [12] and Decon2LS [57]. Decon2LS is an implementation of the THRASH algorithm [58]. We also report on results for the algorithm implemented in OpenMS [4]. This is for reference only, since we use some of the simulation models in our feature detection algorithm as well, which would make benchmarking of the OpenMS algorithm biased.

The algorithms we compared differ heavily in the type and number of parameters they accept. Some require only m/z and retention time range in which to search for features, others require lots of parameters such as confidence cutoffs, bin width or minimum signal-to-noise levels, to name just a few. Parameters are also not always well documented. To achieve a comparison as fair and unbiased as possible, we chose for each algorithms settings that seemed suitable for each simulation run (such as mass resolution and m/z range), but apart from that we decided to stick with the standard parameters and not to further optimize.

### Quality of Simulation

Performing simulations always raises the question whether the simulated data is sufficiently close to reality. In this section, we will demonstrate that our simulations are realistic.

**Figure 7:**
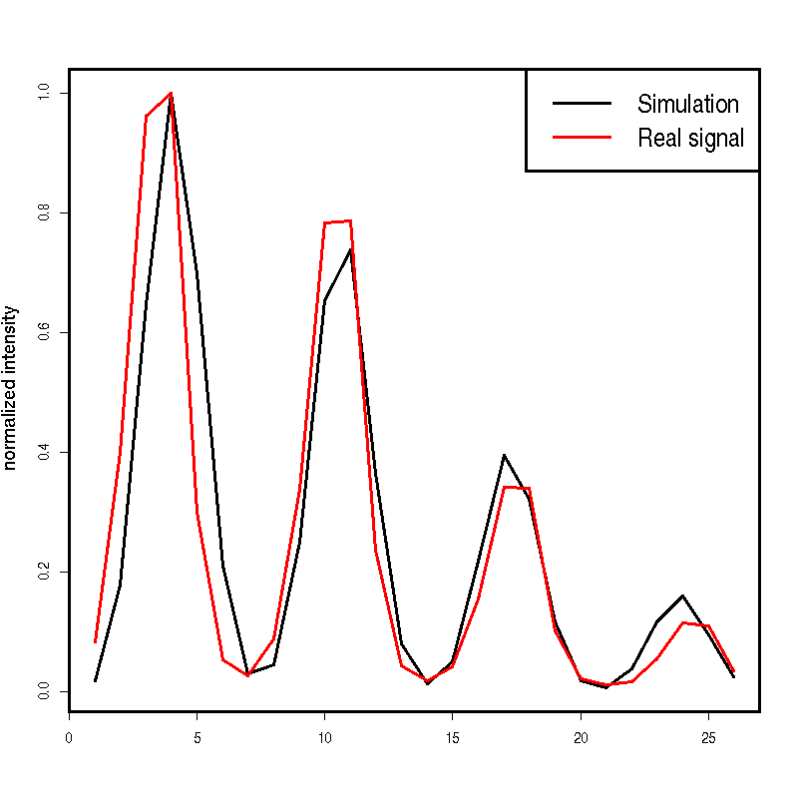
**Assessment of simulation quality**. Comparison of a simulated and a real isotopic pattern. The Pearson correlation between these pattern is high: 0.91.

We already showed that our model of elution peaks and shot noise match real data well (see Fig. 3 and Fig. 5, respectively). To illustrate the quality of our isotopic peak model, we simulated a mixture of standard proteins and generated a real LC-MS run of the same mixture on an ESI-TOF mass spectrometer (microTOF, Bruker Daltonics). Details of the sample preparation are given in [59]. We manually extracted peptide feature signals from the real data set and the simulated LC-MS run and computed Spearman's rank correlation coefficient for three simulated isotopic peak patterns. The correlation coefficients were high, namely 0.91, 0.90 and 0.84. Fig. 7 gives an example. We repeated this experiment with a low resolution LC-MS run. We recorded a mixture of human serum on an ESI iontrap instrument and simulated an LC-MS map of similar resolution. Details of the sample preparation are given in Mayr *et al*. [60]. The correlation between real and simulated isotopic pattern was high (between 0.92 to 0.98). This shows that our isotope distribution model based on the algorithm by Kubinyi [46] and a Gaussian peak shape generates realistic signals.

### Influence of Mass Resolution on Feature Detection

**Figure 8:**
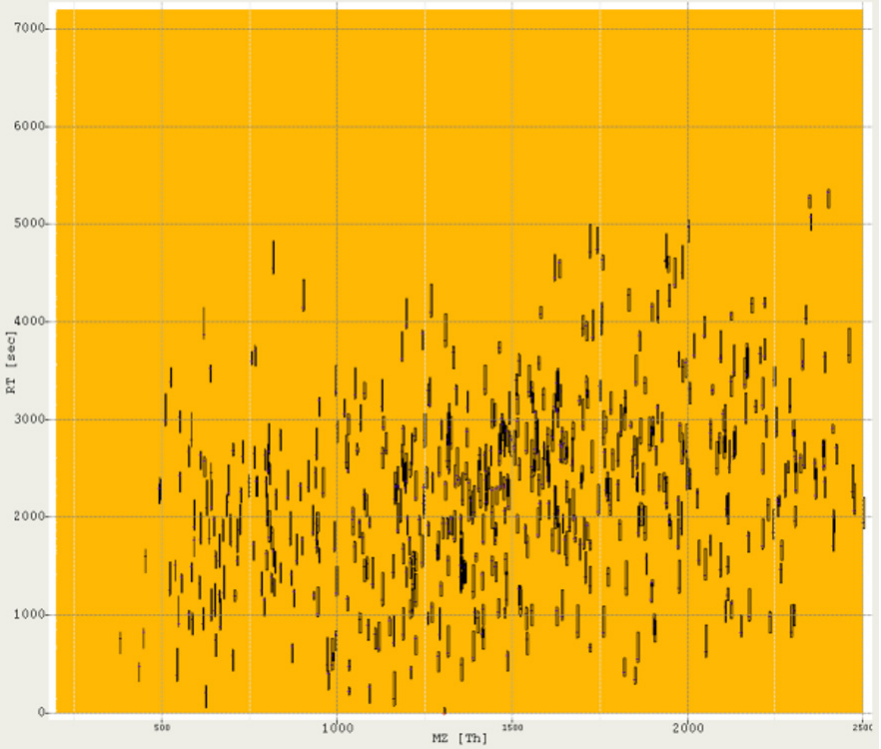
**Simulated LC-MS run of Mouse (Mus musculus) proteins**. The simulated digest of 100 Mouse proteins, for FWHM 0.05. The plot shows the the simulated LC-MS map. Shot noise is yellow and orange/red. The peptide signals are drawn in pink (monoisotopic mass) and with their bounding box in black.

**Figure 9:**
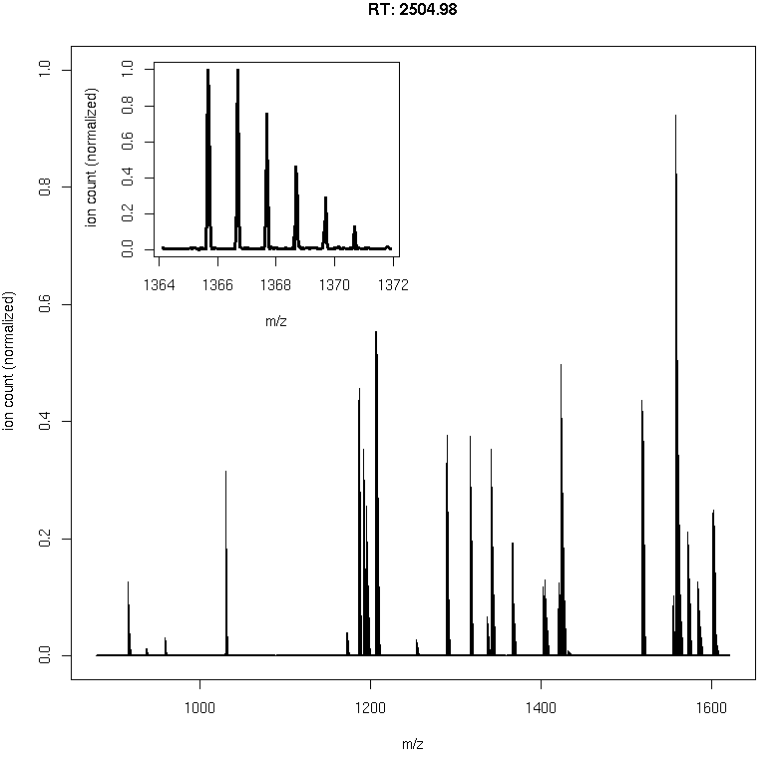
**Simulated mass spectrum taken from Mouse LC-MS map**. A mass spectrum (retention time 2504.98) from the LC-MS map of Mouse proteins.

We downloaded the Mouse IPI protein sequence set (08.04.2008) and randomly selected 100 protein sequences from this set. A tryptic digest and filtering for detectability at a threshold of 0.8 resulted in 820 peptides. The chosen threshold corresponds to a False Discovery Rate of 10%. We opted for this mixture of moderate complexity to avoid a high number of overlapping peptides. Still, manual annotations of all these data sets would be tedious. In our first experiment, our goal was to determine to what extent the performance of current feature detection algorithms depends on the mass resolution of the instrument. We simulated different mass resolutions by changing the FWHM of the peptide isotopic pattern. We generated data sets for FWHM values of 0.05, 0.2, 0.5 and 0.8. A peak FWHM of 0.05 roughly corresponds to an Orbitrap instrument whereas the 0.8 results in spectra similar to typical ion trap measurements. To each data set, we added shot noise with a mean intensity of 150 and a Poisson rate of 450. This noise level was chosen such that all peptide signals would be well above the noise level. The challenge of this benchmark was to detect poorly resolved and possibly overlapping peptide signals. The full result lists are contained in the supplemental material [see Additional file 2] as well as the settings for each feature detection algorithm [see Additional file 3]. We also described our strategy to find suitable parameters for each algorithm [see Additional file 1]. Fig. 8 and Fig. 9 show the simulated LC-MS run for FWHM 0.05 and the spectrum at retention time 2504.98, respectively. All simulated LC-MS maps were uploaded to the PRIDE database  and are available under the accession numbers 8161 to 8168 inclusive.

We noticed early on that each algorithm follows a different strategy. Some algorithms report a lot of potential peptide features even for simple data sets, rather than missing an important signal. The rationale seems to be that it is better to obtains many false positives than to miss a potentially crucial signal. Of course, spurious noise signals can be removed during later stages of the workflow. For instance, by removing signals that do not appear at consistent positions during alignment. Nevertheless, this makes matters unnecessary difficult. In contrast, some algorithms are highly specific but tend to miss poorly resolved signals. Which strategy is best might depend on the specific task to be performed and the complexity of the data.

Furthermore, not every algorithm associates a quality or confidence measure with a feature that could be used as a cutoff. It is therefore not possible to give the classical Receiver Operating Characteristic curves frequently used when comparing signal detection methods. Consequently, we decided to give the results in terms of the False Discovery Rate (FDR) and True Positive Rate (TPR). We approximate the FDR by

$$\text{FDR}=\frac{FalsePositives}{TruePositives+FalsePositives}$$

and compute the TPR as

$$\text{TPR}=\frac{TruePositives}{TruePositives+FalseNagative}.$$

We count a peptide signal as detected correctly if the algorithm found a feature with the correct monoisotopic m/z (with a tolerance of 0.8 m/z) and an estimated bounding box within the true signal bounding box. It happens frequently that an algorithm splits a feature eluting over a longer period of time into several parts, i.e., loses track of the elution peak. In this case, we counted only one true positive hit for this feature but did not count the remaining features as incorrect hits. Table 1 shows the results of this experiment. All algorithms recover most of the peptide signals at high mass resolutions but the True Positive Rate decreases for all algorithms with the resolution. SpecArray performs best on the high resolution data but with decreasing performance at lower resolutions. We would like to emphasize that SpecArray performs very well out-of-the-box, i.e., without parameter tuning, whereas other algorithms required a filtering of signals. Our algorithm, implemented using OpenMS, seems to be less affected by a poor mass resolution but fails to detect some signals even at high resolutions.

**Table 1:** False Discovery and True Positive Rates for changing mass resolutions

FWHM		Superhirn	msInspect	OpenMS	MZmine	SpecArray	Decon2LS
0.05	TPR/FDR	0.88/0.57	0.96/0.46	0.92/0.27	0.78/0.87	0.99/0.05	0.80/0.64
0.2	TPR/FDR	0.68/0.50	0.32/0.97	0.96/0.05	0.71/0.83	0.97/0.41	0.82/0.78
0.5	TPR/FDR	0.10/0.72	0.80/0.98	0.95/0.02	0.33/0.68	0.94/0.54	0.68/0.49
0.8	TPR/FDR	0.003/0.50	0.87/0.98	0.84/0.12	0.03/0.31	0.92/0.36	0.49/0.34

False Discovery and True Positive Rates on four data sets with changing mass resolutions.

We also note that some algorithms, especially msInspect and Decon2LS, compute huge numbers of false positives and consequently, their False Discovery Rates are poor. On the other hand, both algorithms find almost all true signals, especially on the high resolution data set.

**Figure 10:**
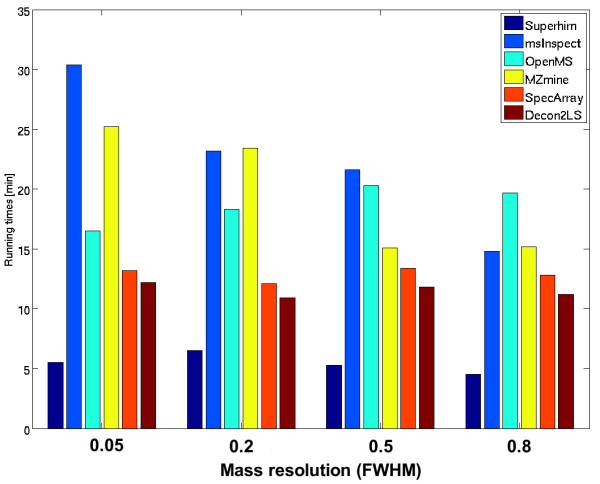
**Running times of the peptide feature detection algorithms**. Running times of all feature detection algorithms on the four data sets with different mass resolutions. Although the running times are comparable, the software Superhirn is the fastest.

Fig. 10 displays the running times of all algorithms on each data set. The time measurements were performed on a 3.2 GHz Intel Xeon CPU with 3 GB memory running Debian or Windows Server 2003 R2 (in the case of Decon2LS, mzMine and msInspect). Note that the running times of Decon2LS and MZmine are approximate results only, since both tools are GUI-based and therefore do not allow direct time measurements. Superhirn stands out as the fastest algorithm, whereas all other tools yield similar running times. Decon2LS is a bit slower than the rest, but not significantly. To summarize, different algorithms have different strengths: some recover nearly all true signals even under poor conditions but at the expense of large numbers of false positive hits. One might argue that many of this false positive signals could be removed by removing features of low intensity or of unlikely masses. But this clearly has its disadvantages if we examine complex mixtures with large dynamic ranges and many compounds at low intensities.

### Influence of Chromatographic Conditions

In this experiment, we tested if the algorithms could deal with noisy elution profiles. We simulated three LC-MS runs, one for each predefined chromatographic condition (good, medium and poor) but kept the mass resolution in terms of the FWHM constant at 0.05. If a peak elution profile gets noisy, we expected most algorithms to lose track of the isotopic pattern over time or maybe even not to detect it at all. Table 2 shows the results of this experiment, again in terms of False Discovery and True Positive Rate.

**Table 2:** False Discovery and True Positive Rates under changing chromatography conditions

condition		Superhirn	msInspect	OpenMS	MZmine	SpecArray	Decon2LS
good	TPR/FDR	0.98/0.87	0.97/0.85	0.85/0.20	0.85/0.19	0.98/0.05	0.72/0.38
medium	TPR/FDR	0.99/0.87	0.98/0.84	0.92/0.23	0.92/0.28	0.99/0.05	0.81/0.64
poor	TPR/FDR	0.98/0.86	0.98/0.84	0.92/0.16	0.91/0.20	0.99/0.05	0.79/0.48

False Discovery and True Positive Rates on three data sets under changing chromatography conditions.

The performance of most algorithms remains stable across chromatographic conditions. There are only two algorithms whose performance lags behind if the elution peaks become noisier, OpenMS and MZmine. The first simulated run with good column conditions contains many overlapping isotopic pattern, and OpenMS is not able to separate strongly overlapping signals. Furthermore, OpenMS uses a Gaussian model to fit the elution curve of a feature and discards features having a poor probability under this model. Obviously, this dampens the performance of OpenMS in this experiment. MZmine does not perform well on high resolution data, as shown in the previous section. This might be due to unfavorable parameter settings. The False Discovery Rate of SpecArray increases slightly at poorer chromatography conditions. All other algorithms are not affected. Note that Decon2LS is not affected by changes in the chromatographic condition since this tool detects isotopic patterns in a scanwise matter and does not take the elution profile into consideration.

### Metabolites

Finally, we tested to what extent current peptide feature detection algorithms can discriminate between peptide signals and signals of other sample compounds. To this end, we generated an LC-MS map consisting of 360 metabolites, but no peptides. These metabolites represent a random subset of compounds from the Human Metabolome Database (accessed 11 March 2008). For each metabolite, we computed its isotopic distribution and placed it at a randomly-determined retention time in the LC-MS map. We modeled the elution profile using a Gaussian function.

Table 3 shows the results of this experiment. The row labelled with PF indicates the percentage of metabolite compounds declared as peptide feature by the algorithm. The second row gives the total number of features reported. As we can see, most algorithms are not able to distinguish between peptide isotopic pattern and metabolite signals. This is probably not surprising as peptides and metabolites exhibit similar isotopic patterns. To illustrate this fact, we drew 2875 metabolites at random from the Human Metabolome Database and computed their isotopic peak profile. After this, we estimated the isotopic peak profile for a peptide of the same mass using the method of *averagines *[61]. This method is used by most feature detection algorithms developed so far.

**Table 3:** Percentage of metabolites declared as features

	Superhirn	msInspect	OpenMS	MZmine	SpecArray	Decon2LS
PF (%)	98.07	99.17	92.56	80.44	98.90	99.17
# features	655	5885	813	283	719	3722

Percentage of metabolites declared as features. We also report the number of features detected in total.

We computed the Pearson correlation coefficient for both, metabolite and peptide isotopic profiles. The lowest correlation is 0.95, which is still high. But this means that current algorithms that try to detect peptide signals using the averagine method will only poorly be able to distinguish peptides from other biomolecules.

This problem might not be grave. If we simply search for signals that discriminate between two conditions e.g. control and disease, it might at first not be that important whether this signal is caused by a peptide or a metabolite. But it is a fact that users have to keep in mind: most feature detection algorithms detect a lot of features in a real world data set, many more than are sequenced. This has usually been attributed to the fact that the data dependent acquisition process is a semi-random sampling of sample compounds and many peptides will never be identified. But users need to be aware that not all detected features will be caused by peptides, but also by other biomaterials including metabolites.

## Discussion

*LC-MSsim *simulates mass spectrometry experiments with a wide range of instrument settings and column performances. There are some ways this software could be improved. To give an example, we trained our SVM predictor for the detectability of peptides on data obtained from MS/MS identifications. That is, our model actually predicts whether a peptide is detected *and *identified using MS/MS. But we use it to predict whether a peptide occurs at all in an LC-MS data set or not. This is clearly a less stringent criterion since not all peptides visible in a mass spectrum will be identified by MS/MS.

It would also be interesting to test another important class of LC-MS data analysis algorithms, namely alignment methods. There is a similar diversity of approaches [62] as for feature detection algorithms and it would be highly beneficial to the computational proteomics community to know about their individual strengths and weaknesses. The next step, as already mentioned in the introduction, would be to test full data analysis pipelines for accuracy of quantification, robustness in the presence of noise and contaminants, etc. Obviously, finding good parameters for each and every pipeline will become even more difficult than it was already in this smaller study. It might be a good idea to compile a benchmark data set consisting of some real and manually annotated LC-MS runs, complemented by a large number of simulated runs. This would be an ideal testing ground for the proteomics community to compare and assess different analysis methods.

To summarize, our aim was not to develop a simulation capturing all physical aspects of an LC-MS experiment. This is hard since not all these aspects are entirely understood. But our aim was to develop a tool which yields benchmark data that are sufficiently close to reality. Furthermore, we tried to keep the source code as modular as possible such that the community can adopt it or add new ideas and simulation models.

## Conclusion

We presented *LC-MSsim*, a simulation software for LC-ESI-MS spectra. Our software contains predictors for peptide retention time and detectability as well as models for charge distribution, peak shapes and isotopic intensity distributions. It has already proved to be valuable for in-house studies and we make it publicly available in the hope that it will be useful to the wider community.

*LC-MSsim *is implemented as an add-on to the OpenMS C++ software library and available for free under an open source license (LGPL). Both OpenMS and *LC-MSsim *can be downloaded from the sourceforge software repository. From a software engineering point of view, *LC-MSsim *is an example how mass spectrometry-related software can easily be built using the OpenMS library.

In this work, we demonstrated the versatility of *LC-MSsim *for the benchmarking of peptide feature detection algorithms. This is a difficult task on real LC-MS data since there is no clearly defined ground truth in this case. We were able to probe the capabilities of currently available algorithms to a deeper extent than previously possible.

## Availability and Requirements

*LC-MSsim *runs under Linux and Windows (using the MingGW compiler). Sourcecode is available from . Installation instructions can be found at . The software depends on several data structures in the OpenMS software library which can be downloaded at .

## List of abbreviations used

FDR: False-Discovery-Rate; FWHM: Full-Width-At-Half-Maximum; LC-MS: Liquid Chromatography coupled to Mass Spectrometry; LGPL: Lesser GNU Public License. Available at ; libSVM: An integrated software for support vector classification, available at ; MudPIT: Multidimensional Protein Identification Technology, it combines 2D chromatography, i.e. two coupled columns, with a mass spectrometer; POBK: Paired Oligo-Border Kernel; SVM: Support Vector Machine; TPR: True Positive Rate.

## Authors' contributions

O.S.T. implemented the simulator, performed the experiments and wrote the manuscript. N.P. and O.K. devised the retention time and detectability prediction. C.G. developed the peak elution profiles and the isotopic peak model. K.R. conceived and initiated the project, provided feedback and directions on the results and the design of the simulator. All authors have read and approved this manuscript.

## Supplementary Material

Additional file 1 **Our strategy to choose the feature detection parameter.** Information about the versions of the tested software tools and their parameter sets. Click here for file

Additional file 2 **Feature detection parameter.** The parameter settings of the different algorithms we compared. Click here for file

Additional file 3 **Results of the feature detection algorithms.** Result lists of the feature detection algorithms executed on our benchmark data. Click here for file

## References

[B1] Mann M, Aebersold R (2003). Mass spectrometry-based proteomics. Nature 422.

[B2] Nesvizhskii AI, Vitek O, Aebersold R (2007). Analysis and validation of proteomic data generated by tandem mass spectrometry. Nat Meth.

[B3] MacCoss M, Matthews DE (2005). Quantitative MS for proteomics: Teaching a new dog old tricks. Anal Chem.

[B4] Schulz-Trieglaff O, Hussong R, Gröpl C, Hildebrandt A, Reinert K, Speed TP, Huang H (2007). A fast and accurate algorithm for the quantification of peptides from LC-MS data. Research in Computational Molecular Biology, 11th Annual International Conference, RECOMB 2007, Oakland, CA, USA, April 21–25, 2007, Proceedings, of Lecture Notes in Computer Science.

[B5] Hoopmann M, Finney G, MacCoss M (2007). High-Speed Data Reduction, Feature Detection, and MS/MS Spectrum Quality Assessment of Shotgun Proteomics Data Sets Using High-Resolution Mass Spectrometry. Analytical Chemistry.

[B6] Du P, Sudha R, Prystowsky MB, Angeletti RH (2007). Data reduction of isotope-resolved LC-MS spectra. Bioinformatics.

[B7] Prakash A, Mallick P, Whiteaker J, Zhang H, Paulovich A, Flory M, Lee H, Aebersold R, Schwikowski B (2006). Signal Maps for Mass Spectrometry-based Comparative Proteomics. Mol Cell Proteomics.

[B8] Lange E, Gröpl C, Schulz-Trieglaff O, Leinenbach A, Huber C, Reinert K (2007). A geometric approach for the alignment of liquid chromatography mass spectrometry data. Bioinformatics.

[B9] Prince J, Marcotte E (2006). Chromatographic Alignment of ESI-LC-MS Proteomics Data Sets by Ordered Bijective Interpolated Warping. Analytical Chemistry.

[B10] Listgarten J, Emili A (2005). Statistical and computational methods for comparative proteomic profiling using liquid chromatography-tandem mass spectrometry. Mol Cell Proteomics.

[B11] Bellew M, Coram M, Fitzgibbon M, Igra M, Randolph T, Wang P, May D, Eng J, Fang R, Lin CW, Chen J, Goodlett D, Whiteaker J, Paulovich A, McIntosh M (2006). A suite of algorithms for the comprehensive analysis of complex protein mixtures using high-resolution LC-MS. Bioinformatics.

[B12] Katajamaa M, Orešič M (2005). Processing methods for differential analysis of LC/MS profile data. BMC Bioinformatics.

[B13] Kohlbacher O, Reinert K, Gröpl C, Lange E, Pfeifer N, Schulz-Trieglaff O, Sturm M (2007). TOPP-the OpenMS proteomics pipeline. Bioinformatics.

[B14] Mueller LN, Rinner O, Schmidt A, Letarte S, Bodenmiller B, Brusniak MY, Vitek O, Aebersold R, Müller M (2007). SuperHirn – a novel tool for high resolution LC-MS-based peptide/protein profiling. Proteomics.

[B15] Mueller LN, Brusniak MY, Mani DR, Aebersold R (2008). An Assessment of Software Solutions for the Analysis of Mass Spectrometry Based Quantitative Proteomics Data. Journal of Proteome Research.

[B16] Piening B, Wang P, Bangur C, Whiteaker J, Zhang H, Feng LC, Keane J, Eng J, Tang H, Prakash A, McIntosh M, Paulovich A (2006). Quality Control Metrics for LC-MS Feature Detection Tools Demonstrated on Saccharomyces cerevisiae Proteomic Profiles. Journal of Proteome Research.

[B17] Thompson JD, Plewniak F, Poch O (1999). BAliBASE: a benchmark alignment database for theevaluation ofmultiple alignment programs. Bioinformatics.

[B18] Julie D, Thompson RR, Patrice Koehl, Poch O (2005). BAliBASE 3.0: Latest developments of the multiplesequence alignmentbenchmark. Proteins: Structure, Function, and Bioinformatics.

[B19] Edgar RC (2004). MUSCLE: multiple sequence alignment with high accuracy andhighthroughput. Nucleic Acids Research.

[B20] Gardner P, Giegerich R (2004). A comprehensive comparison of comparative RNA structure prediction approaches. BMC Bioinformatics.

[B21] Desiere F, Deutsch E, Nesvizhskii A, Mallick P, King N, Eng J, Aderem A, Boyle R, Brunner E, Donohoe S, Fausto N, Hafen E, Hood L, Katze M, Kennedy K, Kregenow F, Lee H, Lin B, Martin D, Ranish J, Rawlings D, Samelson L, Shiio Y, Watts J, Wollscheid B, Wright M, Yan W, Yang L, Yi E, Zhang H, Aebersold R (2004). Integration with the human genome of peptide sequences obtained by high-throughput mass spectrometry. Genome Biology.

[B22] Klimek J, Eddes J, Hohmann L, Jackson J, Peterson A, Letarte S, Gafken P, Katz J, Mallick P, Lee H, Schmidt A, Ossola R, Eng J, Aebersold R, Martin D (2007). The Standard Protein Mix Database: A Diverse Data Set To Assist in the Production of Improved Peptide and Protein Identification Software Tools. Journal of Proteome Research.

[B23] Prince JT, Carlson MW, Wang R, Lu P, Marcotte EM (2004). The need for a public proteomics repository. Nat Biotech.

[B24] Bodenmiller B, Malmstrom J, Gerrits B, Campbell D, Lam H, Schmidt A, Rinner O, Mueller LN, Shannon PT, Pedrioli PG, Panse C, Lee HK, Schlapbach R, Aebersold R (2007). PhosphoPep[mdash]a phosphoproteome resource for systems biology research in Drosophila Kc167 cells. Mol Syst Biol.

[B25] Jones P, Cote RG, Martens L, Quinn AF, Taylor CF, Derache W, Hermjakob H, Apweiler R (2006). PRIDE: a public repository of protein and peptide identifications for the proteomics community. Nucl Acids Res.

[B26] Coombes KR, Koomen J, Baggerly KA, Morris JS, Kobayashi R (2005). Understanding the Characteristics of Mass Spectrometry Data Through the Use of Simulation. Cancer Informatics.

[B27] Wong JWH, Downard KM (2005). Performance of the computer algorithm COMPLX for the detection of protein complexes in the mass spectra of simulated biological mixtures. Journal of Mass Spectrometry.

[B28] ExPASy: Isotopident. http://education.expasy.org/student_projects/isotopident/htdocs/.

[B29] ProteinProspector (MS-Isotope). http://prospector.ucsf.edu/.

[B30] Meek JL (1980). Prediction of Peptide Retention Times in High-Pressure Liquid Chromatography on the Basis of Amino Acid Composition. PNAS.

[B31] Petritis K, Kangas LJ, Yan B, Monroe ME, Strittmatter EF, Qian WJ, Adkins JN, Moore RJ, Xu Y, Lipton MS, Camp DG, Smith RD (2006). Improved peptide elution time prediction for reversed-phase liquid chromatography-MS by incorporating peptide sequence information. Anal Chem.

[B32] Krokhin OV (2006). Sequence-specific retention calculator. Algorithm for peptide retention prediction in ion-pair RP-HPLC: application to 300- and 100-A pore size C18 sorbents. Anal Chem.

[B33] Klammer A, Yi X, MacCoss M, Noble W (2007). Improving Tandem Mass Spectrum Identification Using Peptide Retention Time Prediction across Diverse Chromatography Conditions. Analytical Chemistry.

[B34] Pfeifer N, Leinenbach A, Huber CG, Kohlbacher O (2007). Statistical learning of peptide retention behavior in chromatographic separations: a new kernel-based approach for computational proteomics. BMC Bioinformatics.

[B35] Mallick P, Schirle M, Chen SS, Flory MR, Lee H, Martin D, Ranish J, Raught B, Schmitt R, Werner T, Kuster B, Aebersold R (2007). Computational prediction of proteotypic peptides for quantitative proteomics. Nat Biotech.

[B36] Tang H, Arnold RJ, Alves P, Xun Z, Clemmer DE, Novotny MV, Reilly JP, Radivojac P (2006). A computational approach toward label-free protein quantification using predicted peptide detectability. Bioinformatics.

[B37] Sturm M, Bertsch A, Groepl C, Hildebrandt A, Hussong R, Lange E, Pfeifer N, Schulz-Trieglaff O, Zerck A, Reinert K, Kohlbacher O (2008). OpenMS – An open-source software framework for mass spectrometry. BMC Bioinformatics.

[B38] Schölkopf B, Smola AJ, Williamson RC, Bartlett PL (2000). New Support Vector Algorithms. Neural Computation.

[B39] Sanders W, Bridges S, McCarthy F, Nanduri B, Burgess S (2007). Prediction of peptides observable by mass spectrometry applied at the experimental set level. BMC Bioinformatics.

[B40] Vapnik VN (1995). The nature of statistical learning theory.

[B41] Wu T, Lin C, Weng R (2003). Probability estimates for multi-class classification by pairwise coupling.

[B42] Chang CC, Lin CJ (2001). LIBSVM: a library for support vector machines.

[B43] Iavarone AT, Jurchen JC, Williams ER (2000). Effects of solvent on the maximum charge state and charge state distribution of protein ions produced by electrospray ionization. Journal of the American Society for Mass Spectrometry.

[B44] Konermann L (2007). A Minimalist Model for Exploring Conformational Effects on the Electrospray Charge State Distribution of Proteins. Journal of Physical Chemistry B.

[B45] Schnier PD, Gross DS, Williams ER (1995). On the Maximum Charge State and Proton Transfer Reactivity of Peptide and Protein Ions Formed By Electrospray Ionization. Journal of the American Society for Mass Spectrometry.

[B46] Kubinyi H (1991). Calculation of Isotope Distributions in Mass Spectrometry. A Trivial Solution for a Non-Trivial Problem. Anal Chim Acta.

[B47] Grushka E (1972). Characterization of exponentially modified Gaussian peaks in chromatography. Anal Chem.

[B48] Li J (2002). Comparison of the capability of peak functions in describing real chromatographic peaks. Journal of Chromatography A.

[B49] Naish P, Hartwell S (1988). Exponentially Modified Gaussian functions: A good model for chromatographic peaks in isocratic HPLC?. Chromatographia.

[B50] R Sarpeshkar TD, Mead CA (1993). White noise in MOS transistors and resistors. IEEE Circuits Devices Mag.

[B51] van Etten WC (2006). Poisson Processes and Shot Noise. Introduction to Random Signals and Noise.

[B52] Anderle M, Roy S, Lin H, Becker C, Joho K (2004). Quantifying reproducibility for differential proteomics: noise analysis for protein liquid chromatography-mass spectrometry of human serum. Bioinformatics.

[B53] Du P, Stolovitzky G, Horvatovich P, Bischoff R, Lim J, Suits F (2008). A Noise Model for Mass Spectrometry Based Proteomics. Bioinformatics.

[B54] Shin H, Koomen J, Baggerly K, Markey M (2004). Towards a noise model of MALDI TOF spectra. American Association for Cancer Research (AACR) advances in proteomics in cancer research, Key Biscayne, FL.

[B55] Wishart DS, Tzur D, Knox C, Eisner R, Guo AC, Young N, Cheng D, Jewell K, Arndt D, Sawhney S, Fung C, Nikolai L, Lewis M, Coutouly MA, Forsythe I, Tang P, Shrivastava S, Jeroncic K, Stothard P, Amegbey G, Block D, Hau DD, Wagner J, Miniaci J, Clements M, Gebremedhin M, Guo N, Zhang Y, Duggan GE, MacInnis GD, Weljie AM, Dowlatabadi R, Bamforth F, Clive D, Greiner R, Li L, Marrie T, Sykes BD, Vogel HJ, Querengesser L (2007). HMDB: the Human Metabolome Database. Nucl Acids Res.

[B56] Li Xj, Yi EC, Kemp CJ, Zhang H, Aebersold R (2005). A Software Suite for the Generation and Comparison of Peptide Arrays from Sets of Data Collected by Liquid Chromatography-Mass Spectrometry. Mol Cell Proteomics.

[B57] NCRR Proteomics Resource at PNNL Decon2LS. http://ncrr.pnl.gov/software/.

[B58] Horn DM, Zubarev RA, McLafferty FW (2000). Automated reduction and interpretation of high resolution electrospray mass spectra of large molecules. Journal of the American Society for Mass Spectrometry.

[B59] Schley C, Swart R, Huber C (2006). Capillary scale monolithic trap column for desalting and preconcentration of peptides and proteins in one- and two-dimensional separations. J Chromatogr A.

[B60] Mayr BM, Kohlbacher O, Reinert K, Sturm M, Gröpl C, Lange E, Klein C, Huber C (2006). Absolute Myoglobin Quantitation in Serum by Combining Two-Dimensional Liquid Chromatography-Electrospray Ionization Mass Spectrometry and Novel Data Analysis Algorithms. J Proteome Res.

[B61] Senko M, Beu S, McLafferty F (1995). Determination of Monoisotopic Masses and Ion Populations for Large Biomolecules from Resolved Isotopic Distributions. Journal of the American Society for Mass Spectrometry.

[B62] America AHP, Cordewener JHG (2008). Comparative LC-MS: A landscape of peaks and valleys. Proteomics.

