# Supplementary material for: LC-MSsim – a simulation software for liquid chromatography mass spectrometry data
Source: BMC Bioinformatics. 2008 Oct 8;9:423. doi: 10.1186/1471-2105-9-423 (PMC2577660; doi:10.1186/1471-2105-9-423)
Supplement: Additional file 1 — Our strategy to choose the feature detection parameter. Information about the versions of the tested software tools and their parameter sets. [file 1471-2105-9-423-S1.pdf]

Supplementary Material

Parameter sets for feature selection tools

Several of the tools that we tested depend a lot on suitable parameters. Nevertheless, most parameters are not well documented. On the other hand, some tools work "out-of-the-box" and do not offer any parameters that could be adopted.

We chose the parameter settings for each tool as described below. Three tools (msInspect, Decon2LS and Mzmine) still produced large numbers of false positives, as stated below. We therefore removed for each of these three tools all peptide features with an intensity below the first quantile. The first quantile is the value such that 25% of all feature intensities are below it and the rest above.

msInspect: This tools does not have any parameters besides m/z and rt ranges searched. Each isotope pattern is scored using the Kullback-Leibler (KL) distance between an averagine model and the true peak intensities. A KL distance of zero indicates a perfect match between averagine model and signal and thus a high-quality peptide feature. To find a suitable cutoff for this score, we executed msInspect on a complex LC-MS run, a digest of Halobacterium NRC-1 proteins recorded on a API QSTAR Pulsar I instrument (downloaded from the PeptideAtlas database, ref: Pae000245, data set # 25, http://www.peptideatlas.org/).

We manually annotated this data set and choose 20 intense and well-resolved peptide signals to determine a suitable cutoff. We choose a cutoff for the KL distance of 0.8 such that 80% of the annotated features were detected. Applying this filtering threshold to the whole data set, the number of detected features was reduced from 3366 to 2608 features.

Decon2LS: This tool offers plenty of parameters, some are documented and some are not. According to the authors (personal communication), the fit intensity and the fit score threshold should have significant influence on the result. However, we made the experience that the fit intensity threshold has not much influence on the result. Consequently, we optimized the fit score threshold (a distance measure between 0 and 1, where 0 is best) in the same way as the KL distance described above.

We choose a cutoff for the fit score of 0.2 which resulted in 85% annotated features recovered. The overall number of features was reduced from 19603 to 14592.

MZmine: This software offers different peak detection strategies. We used the "Recursive threshold peak detecter" algorithm. Furthermore, the software has several parameters that influence the feature detection process. This process consists of two

parts: a peak detection and a de-isotoping step. Peak detection is influenced by parameters such as bin sizes, min intensity etc. The de-isotoping step groups peaks into isotopic pattern, estimates a charge and removes incomplete isotope pattern and single peaks. We contacted the developers of Mzmine and asked them which parameters would have significant influence. MZmine does not compute a score and s/n threshold that could be used as single filter criterion. According to the recommendations of the MZmine developers, we adopted the “Chromatographic threshold level” and the “m/z bin size” as well as the noise threshold and minimum peak height which are both given in absolute intensity units.

For the high-resolution data (FWHM 0.02), we choose a set of parameters that recovered 100% of the annotated features from the QSTAR mass spectrometer. With decreasing mass resolution, we relaxed the bin width and the chromatographic threshold level but without satisfying results. In each case, we estimated the noise level as the median intensity on a small m/z interval in empty region (i.e. without peptide signals) of the LC-MS map. We set the minimum peak intensity to the same value.

SpecArray: No parameter changes possible, no manual tuning.

OpenMS + Superhirn : several parameter offered, moderate optimization to achieve trade-off between false positives and false negatives.

Avaibility of software and simulation parameters:

All peptide feature detection algorithms were tested in the version that was available online in January 2008.

In the case of OpenMS, we used a slightly modified version of the 1.0 Release. It can be downloaded from sourceforget using:

svn co https://open-ms.svn.sourceforge.net/svnroot/open-ms/FF10

The installations instructions are the same as for the most recent OpenMS release version (1.2) and can be obtained from www.openms.de (=> follow link "installation").

The parameter settings and peptide feature lists for each feature detection algorithm, as far as they could be stored, are available in the supplement.

The parameter sets are available for download from
http://lcms-sim.sourceforge.net/

The simulation parameter are contained in the svn repository of LC-MSsim:
svn co https://lcms-sim.svn.sourceforge.net/svnroot/lcms-sim lcms-sim

(check subdirectory "ini_files").

The simulated data sets are several GBs large and can be downloaded from the PRIDE database (http://www.ebi.ac.uk/pride/, Accession numbers 8161-8168 incl.).

Ole Schulz-Trieglaff,
trieglaf@cs.fu-berlin.de
